# What is adaptation by natural selection? Perspectives of an experimental microbiologist

**DOI:** 10.1371/journal.pgen.1006668

**Published:** 2017-04-20

**Authors:** Richard E. Lenski

**Affiliations:** 1 Department of Microbiology and Molecular Genetics, Michigan State University, East Lansing, Michigan, United States of America; 2 BEACON Center for the Study of Evolution in Action, Michigan State University, East Lansing, Michigan, United States of America; Dalhousie University, CANADA

## Abstract

Ever since Darwin, the role of natural selection in shaping the morphological, physiological, and behavioral adaptations of animals and plants across generations has been central to understanding life and its diversity. New discoveries have shown with increasing precision how genetic, molecular, and biochemical processes produce and express those organismal features during an individual’s lifetime. When it comes to microorganisms, however, understanding the role of natural selection in producing adaptive solutions has historically been, and sometimes continues to be, contentious. This tension is curious because microbes enable one to observe the power of adaptation by natural selection with exceptional rigor and clarity, as exemplified by the burgeoning field of experimental microbial evolution. I trace the development of this field, describe an experiment with *Escherichia coli* that has been running for almost 30 years, and highlight other experiments in which natural selection has led to interesting dynamics and adaptive changes in microbial populations.

## Evolution, natural selection, and genetics

The fields of biology and evolution have come a long way since Charles Darwin published *The Origin of Species* in 1859. Nonetheless, Darwin is celebrated for the big ideas that he got right, including descent with modification and adaptation by natural selection. The former refers broadly to the fact that evolution has occurred such that organisms living today are different from their ancestors. Natural selection is the evolutionary process that explains the match, or fit, between features of organisms and the environments where they live.

Jean-Baptiste Lamarck and other natural philosophers had previously put forward the idea of evolution in the general sense of descent with modification. And Alfred Russel Wallace, a younger contemporary of Darwin, independently came up with the concept of adaptation by natural selection. Neither space nor expertise allows me to do justice to the history of these ideas, except to note that Darwin is better known today than Wallace because Darwin brought to bear an extraordinary range of relevant evidence and insights that have, by and large, stood the test of time. When Darwin was rushed at the age of 50 to publish *The Origin* by virtue of Wallace’s discoveries, he produced a 502-page volume rich with insights and details that he called a mere “abstract” of the great book he had intended to publish. Over his remaining years, Darwin published many more books—*The Variation of Animals and Plants Under Domestication* (1868), *The Descent of Man*, *and Selection in Relation to Sex* (1871), and *The Expression of the Emotions in Man and Animals* (1872) among them—that provided further insights and more evidence concerning his core theories of descent with modification and adaptation by natural selection.

Lamarck is now known largely for his theory of the inheritance of acquired characteristics. While Lamarckian inheritance has been soundly rejected as a general theory of biological inheritance, it seems to have foreshadowed certain special cases in biology in which an environmental agent induces an adaptive genetic change. For example, when a lysogenic phage infects a bacterium, the phage’s DNA can integrate into the bacterial chromosome and thereby confer immunity to reinfection by another phage. Similarly, CRISPR/Cas (clustered regularly interspaced short palindromic repeats/CRISPR-associated protein) systems allow bacteria and archaea to incorporate bits of DNA from phages and plasmids that provide immunity against later infections [1]. Cultural evolution in humans also occurs via acquisition from the environment (by learning) and inheritance that is, in that respect, Lamarckian. Certain maternal effects and epigenetic mechanisms are also sometimes said to be Lamarckian. However, these are special cases and different from the general theory that Lamarck proposed, which has been supplanted by modern genetics and molecular biology. Moreover, these quasi-Lamarckian special cases—at least those that confer clear benefits—presumably evolved by the Darwinian process of adaptation by natural selection.

But Darwin, too, got some things wrong. His proposed mechanism for inheritance involved “gemmules” made throughout the body and then concentrated in the reproductive organs, allowing transmission across generations in a rather Lamarckian manner. Darwin also thought the process of evolution was too slow to directly observe. In *The Origin*, he wrote: “We see nothing of these slow changes in progress, until the hand of time has marked the long lapse of ages, and then so imperfect is our view … that we only see that the forms of life are now different from what they formerly were.” This view seems rather surprising, given that *The Origin* began by discussing the process of domestication and using artificial selection as practiced by plant and animal breeders to inform the theory of natural selection. Yet even there, he wrote: “Slow and insensible changes of this kind could never be recognised unless actual measurements or careful drawings of the breeds in question had been made long ago, which might serve for comparison.”

The impact and reach of Darwin’s theories are well reflected in T. H. Huxley’s quip, “How extremely stupid not to have thought of that,” and in the title of a paper by Theodosius Dobzhansky [2]: “Nothing in biology makes sense except in the light of evolution.” However, while zoologists and botanists largely embraced adaptation by natural selection following the rediscovery of Mendelian inheritance and the rise of population genetics leading to the Modern Synthesis, many microbiologists were skeptical of its importance to the organisms they studied. For example, I. M. Lewis [3] wrote, “The subject of bacterial variation and heredity has reached an almost hopeless state of confusion … There are many advocates of the Lamarckian mode of bacterial inheritance, while others hold to the view that it is essentially Darwinian.” As a consequence, Julian Huxley [4] excluded bacteria from the Modern Synthesis in 1942, writing “They have no genes in the sense of accurately quantized portions of hereditary substance …”

That changed the very next year, however, when Salvador Luria and Max Delbrück [5] published their fluctuation test, which showed that mutations in *E*. *coli* that confer resistance to viruses could occur before exposure. That meant that natural selection was responsible for the rise in frequency of the resistant mutants following exposure but not for their mutational origin. The replica-plating experiment of Joshua and Esther Lederberg [6] provided another, even more direct, demonstration of the conceptual distinction between the origin of genetic variants by mutation and the fate of those variants, which depended on selection.

## Evolution observed

**Fig 1:**
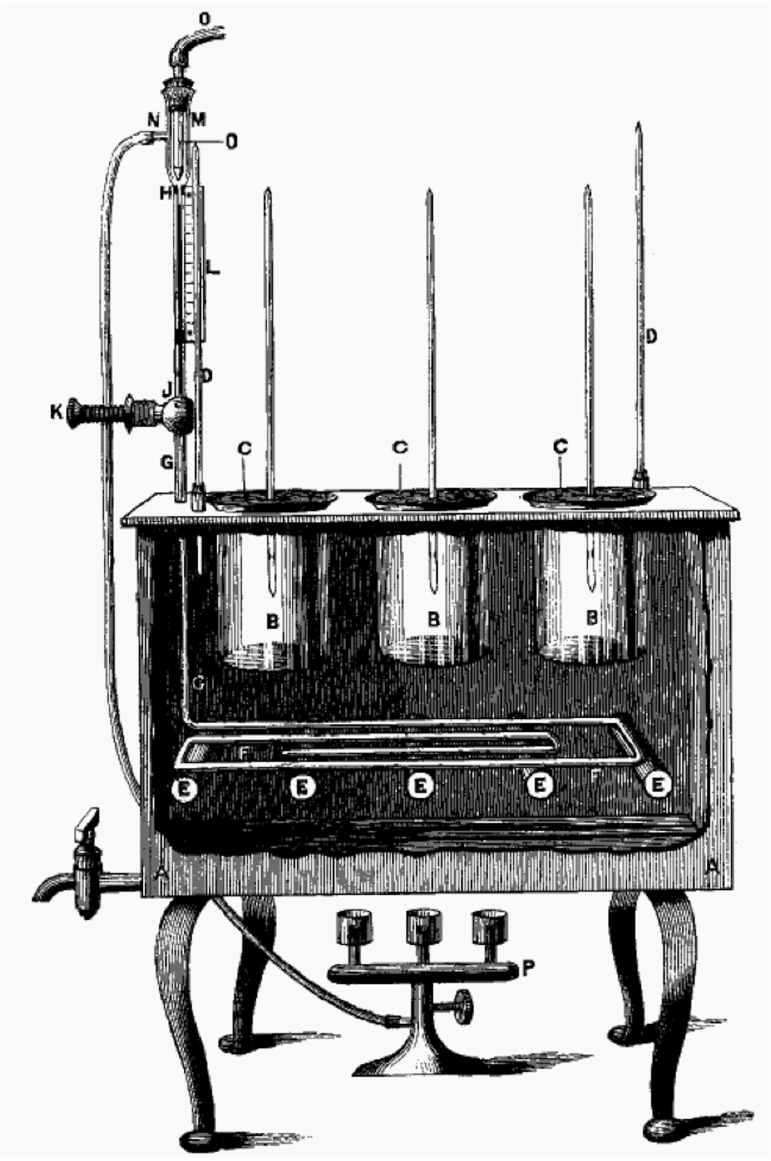
Incubator used in early experiment on adaptation by natural selection. Image from Dallinger (1887), now in the public domain (http://commons.wikimedia.org/w/index.php?curid=10531922).

Though Darwin thought evolution was too slow a process to observe directly, not all of his contemporaries agreed. In particular, William Dallinger put Darwin’s theories to the test in the 1880s. An ordained minister and future president of the Royal Microscopical Society, Dallinger built an incubator in which he cultivated three protozoan species, gradually raising the temperature over several years before an accident ended the experiment [7, 8] (Fig 1). Over time, new strains arose that grew at temperatures lethal to the original strains. One wonders, in retrospect, whether these strains were mutants, representatives of a diverse community present at the outset, or perhaps contaminants, although his account shows the great care with which he ran the experiment and monitored the organisms. In any case, this work showed how one could watch evolution in action using microorganisms. As Dallinger himself put it: “I can only claim for this fragment its suggestiveness, and its possible value as an incentive to treat the lower and minuter forms of life in corresponding manners, and as showing that such work cannot be without value.”

It would be many decades, however, before this value was fully realized. The experiments of Luria, Delbrück, and the Lederbergs had demonstrated that mutation and selection were distinct processes, but their main impact was in genetics, where they set off the revolution that became the field of molecular genetics by showing that microbes were superb models for understanding the physicochemical basis of heredity. Nonetheless, the importance of natural selection for the “minuter forms of life” took hold, albeit tenuously, as the result of key papers in the early 1950s.

Aaron Novick and Leo Szilard had worked on the Manhattan Project before their interests turned to biology. They sought to estimate mutation rates by measuring the rate at which phenotypically defined classes of mutants accumulated in *E*. *coli* populations growing in a chemostat, provided the mutants grew at the same rate as their parents. (If the mutants grew more slowly, as some did, they would reach a mutation–selection balance.) Novick and Szilard [9, 10] saw for a while the expected linear accumulation of mutants, followed by a precipitous drop in their frequency and then a resumption of the linear increase. They hypothesized that the sudden decline in the frequency of the observed mutants reflected an unseen beneficial mutation that arose in the parental background. As the fitter mutant type swept through the population, it displaced the parent strain and the observable mutants derived from the parent. Once the fitter type had become common, then it, too, began to generate measurable numbers of the observable class of mutants. Novick and Szilard tested this hypothesis by competing two strains under the same conditions: one strain bearing the observable neutral mutation isolated before the reversal, and therefore in the parental background, and the other an unmarked strain sampled after the reversal, which was hypothesized to have a beneficial mutation. As predicted, the later strain outcompeted the earlier one, and the same outcome held when the states of the neutral mutation were reversed.

Similar experiments were performed by K. C. Atwood, Lillian Schneider, and Francis Ryan [11, 12], who saw multiple selective sweeps and introduced the term “periodic selection” to describe the phenomenon. Among these early practitioners of experimental evolution, Ryan seems to have been especially smitten by the approach and its implications. In an article titled “Evolution Observed” for *Scientific American* [13], he wrote: “And so the process continued: we obtained successively fitter and fitter types through 7,000 generations. All this time the medium, i.e., the environment, was kept constant … It is sometimes contended that mutations cannot provide the raw material for evolution because they are usually deleterious. But these experiments prove that selection is a powerful force for fixing and perpetuating those rare mutations that do give an advantage.”

These insightful experiments were performed before the physical basis of heredity was known. With the discovery of the double helix by James Watson and Francis Crick in 1953, genetics research became dominated by molecular approaches, and experimental studies of microbial evolution fell largely by the wayside. As a former postdoctoral researcher with George Beadle and Edward Tatum, a mentor of Joshua Lederberg, and an active participant in the management of the Cold Spring Harbor Laboratory, Ryan was well positioned to help keep the fields of evolutionary biology and molecular genetics connected, if not united. Alas, he died in 1963 at the age of just 47.

## Microbial experimental evolution redux

Even as biology became increasingly split between molecular biology and “old-fashioned” studies (including evolutionary biology, ecology, and studies of whole organisms rather than their constituent molecules), some wanderers and visionaries found fertile ground between the two camps. Carl Woese used the molecules of life to reveal the deep history and previously hidden diversity of microbes [14]. Roger Milkman [15] and Robert Selander and Bruce Levin [16] followed the lead of population geneticists in using molecular markers to understand the evolutionary processes that act on contemporary populations of bacteria in nature.

And still others conducted evolution experiments with microbes—sometimes to see what interesting adaptations they could produce, sometimes to better understand the dynamics of adaptation by natural selection. Patricia Clarke, Barry Hall, and Robert Mortlock [17] were leaders in the first group, observing how bacteria could evolve new functions by, for example, constitutively expressing a protein with promiscuous activity on a novel substrate and then adapting the protein to that substrate by subsequent mutations. Using the Qβ bacteriophage, Sol Spiegelman evolved a dramatically shortened RNA genome that could self-replicate in a cell-free medium [18].

On the dynamics front, Lin Chao, Bruce Levin, and Frank Stewart studied the diversification of coevolving phage T7 and *E*. *coli* through successive bouts of resistance and host-range mutations [19]. In a study with the yeast *Saccharomyces cerevisiae*, Charlotte Paquin and Julian Adams showed that nontransitive competitive interactions—where B beats A, and C beats B, but A prevails against C—could lead to long-term declines in fitness, even as each replacement was driven by natural selection [20]. Using different alleles of a core metabolic gene from natural isolates of *E*. *coli*, Daniel Dykhuizen and Daniel Hartl moved them into a common genetic background to test if they affected fitness or were selectively neutral [21].

## Evolution unlimited?

I direct a long-term evolution experiment (LTEE) with *E*. *coli*. Six populations were founded in 1988 from each of two ancestral strains that differ by a neutral marker [22]. There are no plasmids or functional phages, and *E*. *coli* is not naturally transformable, so evolution is strictly asexual. Spontaneous mutations provide all the genetic variation on which natural selection acts. The populations live in a minimal medium with glucose as the limiting resource. Every day, 1% of each population is transferred to a flask containing fresh medium, where the cells grow until they exhaust the glucose and then sit in stationary phase until the next day. The 100-fold regrowth permits ~6.7 cell generations per day. Samples of each population are periodically stored frozen, and where they are available for later study. The frozen samples also allow the populations to be restarted after accidents or disruptions. At this writing, the populations have passed 66,000 generations, and the goal is to continue the experiment far into the future [23].

I had been a postdoctoral researcher with Bruce Levin, building on his work on coevolving bacteria and phage [19, 24]. When I started my lab, I continued working on interactions of bacteria, viruses, and plasmids, asking whether the fitness costs, or tradeoffs, associated with resistance to viruses and antibiotics were fixed or, alternatively, could be ameliorated by compensatory adaptations [25, 26]. However, the interactions were complex and the analyses difficult, so I undertook the LTEE to ask some basic questions about the process of adaptation: (i) What are the dynamics of adaptation by natural selection? Is adaptation invariably slow and gradual? Or are there periods of rapid change and stasis? For how long can fitness increase? (ii) How repeatable is adaptive evolution? Will replicate populations evolve along similar paths? Or will they find different solutions to identical environments? (iii) How are the dynamics of phenotypic and genomic evolution coupled? What functional changes are responsible for the bacteria’s adaptation by natural selection?

### Dynamics of adaptation by natural selection

The dynamics are interesting, and sometimes surprising, in several respects. During the first 2,000 generations or so, the effect sizes of beneficial mutations were large and produced fitness trajectories with step-like dynamics [23, 27]. Over longer periods, the rate of improvement slowed substantially [27, 28]. That trend might suggest that fitness is approaching some upper bound, or asymptote. However, the fitness data are better fit by a simple two-parameter power-law model, which has no asymptote, than by an equally simple hyperbolic model [28]. Moreover, the power-law model predicts fitness levels accurately far into the future using truncated datasets [28]. And a simple dynamical model with clonal interference (i.e., competition between lineages with different beneficial mutations [29]) and diminishing-returns epistasis (i.e., beneficial mutations confer smaller advantages in more-fit than in less-fit backgrounds) generates a power-law relation [28].

### Repeatability of adaptation

Over 50,000 generations, a typical population increased fitness by ~70% relative to the ancestor [28], whereas a typical pair of populations differ from one another by only a few percent [30]. Against this backdrop of predictability, however, some populations stand out in interesting ways. Half of the populations evolved hypermutable phenotypes [31, 32], which led to slightly faster rates of fitness improvement [28, 30]. However, several of those later evolved compensatory changes that reduced their mutability, reflecting the tension between the production of beneficial mutants that are the next big winners and the cost of producing progeny with deleterious mutations [32, 33]. The populations also vary in whether or not they generated stable polymorphisms that sustain diversity within them. One population has two lineages that have coexisted for over 40,000 generations [32, 34]. Their coexistence depends on crossfeeding, in which one lineage is the superior competitor for the exogenously supplied glucose and the other is better at using acetate excreted into the medium [34, 35]. Other LTEE populations have had transiently stable polymorphisms [36], and still others appear to have remained more homogeneous [32], although metagenomic sequencing may reveal previously undetected polymorphisms.

**Fig 2:**
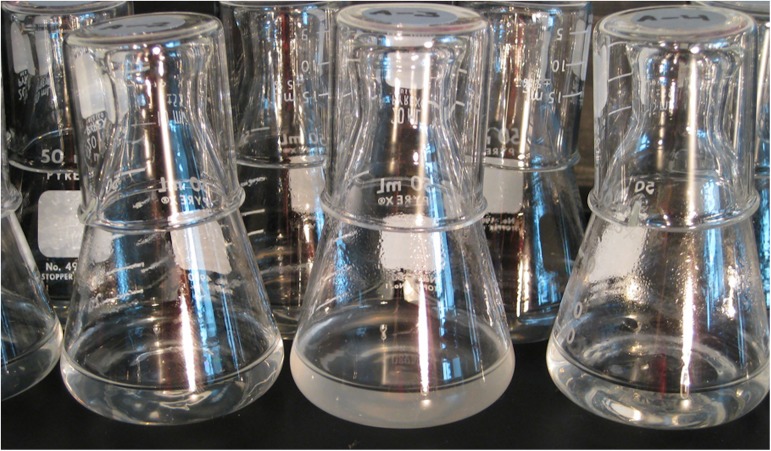
Experimental populations of *E*. *coli*, centered on the population that evolved the ability to use citrate in the LTEE. Photo by Brian Baer and Neerja Hajela, Michigan State University (http://commons.wikimedia.org/w/index.php?curid=4277502).

Most strikingly, one population evolved the ability to grow on citrate at ~31,000 generations [37] (Fig 2), while none of the others have done so even after 66,000 generations. Citrate has been present in the medium throughout the duration of the LTEE, where it serves as a chelating agent. In principle, citrate provides another source of carbon and energy, but one of the defining characteristics of *E*. *coli* as a species is that it cannot take up and use citrate in the presence of oxygen. Each LTEE population has tested billions of mutations over time, so the difficulty of evolving the ability to use citrate does not reflect a scarcity of mutations; moreover, the population that evolved this ability was not hypermutable when it did so [38]. Instead, the difficulty of evolving this ability reflects two issues. First, expression of the relevant transporter protein required a “promoter capture” that involved rearranging nonhomologous DNA segments to produce a new module [38]. Second, even with the new module in place, efficient growth on citrate requires certain other mutations in the genetic background [37–40].

### Coupling of phenotypic and genomic evolution

When the LTEE began, not a single bacterial genome had been sequenced, and for many years whole-genome sequencing was too costly for this project. Nevertheless, by working back from phenotypic changes to candidate genes and using other approaches, some mutations were discovered; and once a mutation was found in one population, that gene was sequenced in the others [41–46]. This approach revealed many examples of parallel evolution at the level of genes, but because of the ad hoc ways that genes of interest were found, it was difficult to assess the global extent of parallelism and the proportion of the accumulated mutations that were beneficial.

In time, though, it became feasible to sequence and analyze complete genomes, including, most recently, 264 clones in total from the 12 independent populations [32]. The data give an extremely strong signal of genomewide parallelism, with over 50% of nonsynonymous mutations that arose in nonhypermutable lineages concentrated in just 2% of the protein-coding genes. Significant parallelism was also seen in the hypermutable lineages, although the signal was much weaker because beneficial mutations were diluted in a larger pool of neutral and weakly deleterious mutations. While there was strong parallelism at the level of genes, there were very few cases where the exact same mutations were found in any two replicate populations. Parallelism at the level of genes, and not at the level of nucleotides, supports the inference that natural selection, rather than mutational hotspots, drove the enrichment of the point mutations. The ratio of nonsynonymous to synonymous mutations, adjusted for the number of sites at risk for each, was >10 over the first 500 generations of the LTEE and has remained >2 even in later generations, providing another strong signal of natural selection [32].

Much work remains to be done to understand the effects of these mutations. A number have been demonstrated to be beneficial by constructing and competing genotypes that differ by specific mutations [47, 48], but how they are beneficial is often unclear. The genes with beneficial mutations include ones that encode proteins with core metabolic and regulatory functions [32]. These genes are likely to have pervasive pleiotropic and epistatic effects, contributing to the difficulty in understanding exactly how mutations in those genes benefit the cells.

## An explosion of experimental evolution

The field of experimental evolution has grown tremendously in recent years. Using the Google Ngram viewer (http://books.google.com/ngrams) for the period from 1948–2008, the word “evolution” has trended gradually upward from ~0.003% to ~0.004%. Although used far less often, the phrase “evolution experiment” showed an ~10-fold increase in use over that period (based on a 10-year running average). It is impossible to do justice to this field here, but several recent reviews that focus on evolution experiments using microbes are available [49–51]. Instead, I highlight a dozen papers that illustrate the wide range of issues being studied.

Several studies have documented the emergence of complex interactions between bacterial genotypes derived from the same ancestral strain. Rainey and Travisano [52] showed that populations of *Pseudomonas fluorescens* rapidly diversified when cultured in static flasks but did not if the flasks were shaken. The diversification occurred because the static flasks generated environmental gradients, which allowed ecotypes with different environmental preferences to flourish. Zambrano et al. [53] starved *E*. *coli* populations and found mutants that could grow while the other cells were dying. Fiegna et al. [54] studied a mutant strain of *Myxococcus xanthus* that could produce spores only by exploiting other strains that made fruiting bodies. From this obligate cheater, they evolved a strain that not only made fruiting bodies and spores on its own but that also was resistant to cheating by its progenitor.

Other studies have examined the evolution of bacteriophages and the role of host–parasite coevolution. Wichman et al. [55] watched two populations of phage ϕX174 evolve at high temperature while growing on a novel host, *Salmonella typhimurium*, and then sequenced the phage genomes. They saw striking parallelism across the replicates, with about half of the mutations that reached high frequency identical at the nucleotide level. Paterson et al. [56] compared the rate of evolution in phage ϕ2 when its *P*. *fluorescens* host was allowed to coevolve and when the host was prevented from evolving by repeatedly restarting it from a stock culture. They found that the phage’s genome evolution was faster and more variable across replicates when its host was coevolving, consistent with Red Queen dynamics. The coevolutionary dynamic between phage λ and *E*. *coli* also enabled Meyer et al. [57] to select phage genotypes that could infect cells using a new receptor, a shift not seen in many decades of previous studies of this interaction.

A different sort of coevolution—one with major health implications—occurs when humans increase antibiotic concentrations in an effort to control bacteria. A study by Lindsey et al. [58] showed that *E*. *coli* populations could sometimes be driven to extinction by raising the concentration quickly, which prevented the bacteria from evolving the high-level resistance they reached when it was raised slowly. By contrast, Baym et al. [59] built arenas where populations of motile *E*. *coli* evolved in a stepwise fashion to grow at progressively higher antibiotic concentrations. Their time-lapse videos provide a striking demonstration of evolution in action (http://vimeo.com/180908160/7a7d12ead6).

**Fig 3:**
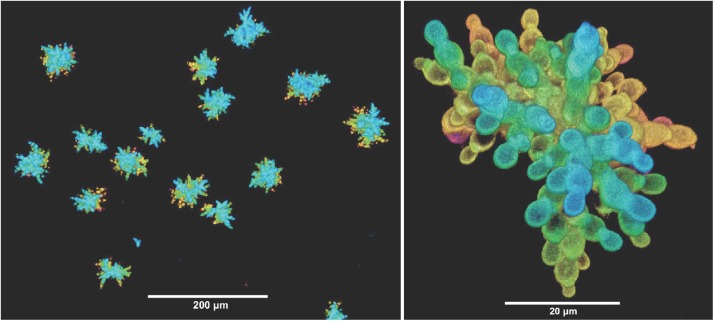
Clusters of “snowflake” yeast that evolved a multicellular life history. Confocal micrographs showing many clusters (left) and one at higher magnification (right). Colors show depth in z-axis. Unpublished images by Shane Jacobeen, Will Ratcliff, and Peter Yunker, Georgia Institute of Technology.

Some studies have used creative selection schemes to generate interesting adaptations. Ratcliff et al. [60] performed centrifugation to select fast-settling *S*. *cerevisiae* and evolved “snowflake” yeast with a multicellular life history (Fig 3), which in turn favors a division of labor between soma and reproductive cells. Most evolution experiments select for mutants that grow faster than their competitors, whereas many real-world applications need strains with higher yields, not faster growth. Bachmann et al. [61] evolved high-yield *Lactococcus lactis* using a water-in-oil emulsion system. Mutants that grew more efficiently had access to the remaining resources within a droplet, thereby preventing takeover by other mutants that grew faster but less efficiently.

New methods for watching the dynamics of genome evolution have also advanced the field. Lang et al. [62] used metagenomic sequencing to study the dynamics of within-population polymorphisms in 40 experimental populations of yeast. Levy et al. [63] used barcodes to track lineages in an evolving yeast population, revealing thousands of beneficial mutations that initially rose in frequency but ultimately were outcompeted by the most-fit lineage.

The studies highlighted in this short review have used microbes, but many other evolution experiments employ flies, mice, and other large organisms [64]. A few evolution experiments have even been performed not in the laboratory but in natural environments [65, 66]. And, of course, many studies of adaptation by natural selection take place without designed experiments, including the extraordinary multidecadal study of Darwin’s finches in the Galápagos by Peter and Rosemary Grant [67], as well as huge swaths of comparative biology [68]. This review only scratches one surface of the body of research on adaptation by natural selection.

## Conclusions

Adaptation by natural selection has been central to biology ever since Darwin presented the idea more than 150 years ago. When coupled to theories of mutation and inheritance, it explains how organisms become fit to their environments. Microbiologists were, on the whole, slower to accept the generality of this theory than those who studied plants and animals. Following critical experiments that disentangled the effects of mutation and selection in microorganisms, and given their short generations and large populations, experimental evolution has become a highly productive approach in microbiology. Some of the experiments test specific hypotheses, while others, like the LTEE, are open-ended and explore broad questions. New technologies enhance the power of experimental evolution, which may in turn provide new opportunities for applied studies in biotechnology and medicine. As evolutionary biology continues to generate fascinating ideas and questions, experimental evolution offers one approach for examining new ideas and questions.
